# MRI Biomarkers for Hand-Motor Outcome Prediction and Therapy Monitoring following Stroke

**DOI:** 10.1155/2016/9265621

**Published:** 2016-09-26

**Authors:** U. Horn, M. Grothe, M. Lotze

**Affiliations:** ^1^Functional Imaging Unit, Department of Diagnostic Radiology and Neuroradiology, University Medicine, University of Greifswald, Greifswald, Germany; ^2^Department of Neurology, University Medicine, University of Greifswald, Greifswald, Germany

## Abstract

Several biomarkers have been identified which enable a considerable prediction of hand-motor outcome after cerebral damage already in the subacute stage after stroke. We here review the value of MRI biomarkers in the evaluation of corticospinal integrity and functional recruitment of motor resources. Many of the functional imaging parameters are not feasible early after stroke or for patients with high impairment and low compliance. Whereas functional connectivity parameters have demonstrated varying results on their predictive value for hand-motor outcome, corticospinal integrity evaluation using structural imaging showed robust and high predictive power for patients with different levels of impairment. Although this is indicative of an overall higher value of structural imaging for prediction, we suggest that this variation be explained by structure and function relationships. To gain more insight into the recovering brain, not only one biomarker is needed. We rather argue for a combination of different measures in an algorithm to classify fine-graded subgroups of patients. Approaches to determining biomarkers have to take into account the established markers to provide further information on certain subgroups. Assessing the best therapy approaches for individual patients will become more feasible as these subgroups become specified in more detail. This procedure will help to considerably save resources and optimize neurorehabilitative therapy.

## 1. The Challenge: Preparing the Field

Stroke continues to be the leading cause for long-term disabilities. Worldwide, about 5 million people who have suffered from a stroke remain permanently impaired [1], leaving a majority of patients with disturbances in the motor abilities [2]. About 75% of those who experience stroke have lingering upper limb impairment [3]. As restoring hand-motor abilities is crucial in improving the patients' daily lives, the efficiency of training strategies is essential. In contrast to lower limb training, which only focuses on pure repetition of gait movements [4], upper limb motor function training needs to combine different aspects of motor abilities to rehabilitate the everyday requirements [5]. Plasticity research has also suggested that repetitions close to the individual output limit improve motor ability more than the number of overall repetitions alone [6]. It is therefore crucial to adjust the therapy to the functional requirements of each patient.

The therapeutic success depends on the amount of lesioned brain resources and the capability of affected systems to adapt to alternative intact resources. In addition, the time after stroke is a relevant factor for plasticity: specifically, the acute and subacute phases after stroke are characterized by augmented plasticity, which can last up to 3 or 4 months [7]. In these early stages, therapeutic intervention may lead to functionally relevant improvements whereas, in the chronic stage, the potential to recover basic functions is limited (e.g., [8, 9]).

**Figure 1:**
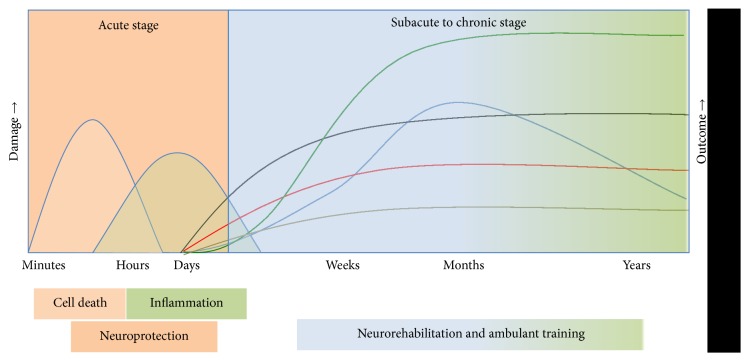
This graph illustrates the need for prognostic assessment tools, especially for the clinically important subacute stage. In the acute stage, cell death and inflammation are associated with worse outcome, and the therapeutic goal therefore is neuroprotection. In the subacute to chronic stage, neurorehabilitation individually improves clinical outcome.

Figure 1 provides an overview of the different stages after stroke for the adult patient.

So far, it has not been possible to properly assess individual recovery processes. Based on clinical presentation alone, it is difficult to estimate which patients will recover their upper limb function [10]. Prognostic measures are needed to identify the individual potential for improvement and to predict the individual outcome after stroke. Imaging techniques can complement the clinical assessment and provide an insight into the patient's individual plasticity processes and offer appropriate therapy. The diagram was modified after [11].

## 2. Outcome Assessment

The definition of a unified motor outcome assessment is difficult and the choice of the outcome parameter is highly dependent on the patient group being investigated: patients with low outcome are not able to perform more demanding tests, while those with high motor outcome show ceiling effects in less demanding tests. There also seems to be a discrepancy between the outcome measurement used in the rehabilitative phase and the real life relevance of this motor performance. Different scores can rate the different abilities recovering over time, for example, measuring strength, aiming, pinch grip, and tapping tasks. For instance, motor training in healthy participants modulates four independent motor abilities: aiming, speed, steadiness, and visuomotor tracking [13]. It would be desirable to represent these four different motor abilities in outcome scores more specifically. Another suggestion for optimizing outcome measurements is to apply an objective measurement for the usage of the affected hand in activity of daily living (ADL), for instance, with accelerometers [14]. Data gained by accelerometers as outcome measures might also solve the problems of interrater variability and the nonparametric distribution of scores. Measuring different outcome scores at once, which are highly associated, causes a multiple comparison problem. To solve this problem, large sample sizes are necessary to differentiate predictors for several performance outcome parameters. Rather than averaging over different scores, a certain parameter which depicts the relevant motor ability most accurately seems desirable.

It is possible to summarize those measures which illustrate the recovery best, for example, by building a composed score with principal component analyses (PCA) [15–17]. However, the composed recovery score does not reveal which component leads to the measured improvement, and the PCA scores are also dependent on the specific data set, which varies with different studies.

For a single patient, the outcome parameters can be set individually, depending on the requirements of and relevance to the patient. In prediction studies, however, it is necessary to assess several abilities which might be relevant to the outcome of the specific group. PCA therefore are able to assess the main effects of the group rather than the individual when several variables might influence the outcome.

## 3. Contributions of Imaging on Neural Substrates of Motor Recovery

Different imaging strategies have been developed addressing functional loss in different stages after stroke.

Specifically, structural imaging has been important in determining the outcome and understanding of functional loss. However, most of these approaches are poorly standardized and usually expert knowledge is required to estimate the exact localization or extent of the damage. There are some approaches simplifying the research methods to assess structural damage of the white matter (e.g., for the pyramidal tract [18]).

In contrast, functional imaging has so far only partially contributed to the understanding of the neural mechanisms of recovered hand-motor function and has gained almost no access to clinical routine over the past decades. We will discuss some problems that the frequently used imaging techniques are facing and what is necessary to overcome those issues.

**Figure 2:**
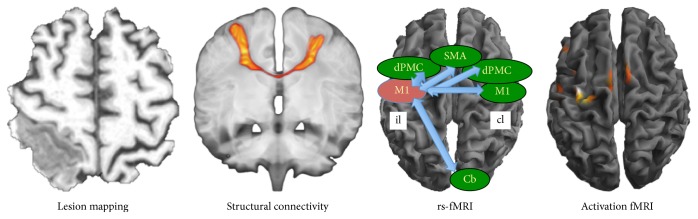
Two structural and two functional imaging methods applied in monitoring and predicting hand-motor outcome after stroke. From left to right: lesion mapping on a T1 weighted imaging dataset; diffusion-weighted imaging measuring structural connectivity, demonstrated here with probabilistic tracking between the bilateral primary motor cortices; resting-state fMRI (rs-fMRI) assessing functional connectivity between different regions of interest (Cb: cerebellar anterior hemisphere; dPMC: dorsal premotor cortex; SMA: supplementary motor area); and activation fMRI during active grip strength task with the affected right hand.

Figure 2 provides an overview of the most frequently used imaging techniques for stroke motor imaging.

### 3.1. Structural Imaging

The clinically most important contribution of MRI to the evaluation of hand-motor outcome is the precise quantification of the damaged neural resources. Particularly, the lesion location and the amount of damage of parts of hand movement representation and their white matter connections are critical parameters assessed with structural imaging (e.g., [19]). Additional impairment (e.g., somatosensory impairment [20]) might decrease the outcome of conventional motor training. It is crucial to add this information in prognostic decisions to allow for more specified training. In addition, some patients with certain lesion locations show extremely good functional recovery (e.g., anterior cerebellar hemisphere lesion), whereas others have almost no recovery potential (e.g., with brain stem or cerebellar vermis lesions). The evaluation of motor impairment, outcome, and therapy approaches is usually based on the experience of the neurologist. However, it usually is difficult to predict the clinical outcome based only on this information. Apart from the individual clinical information like age, concomitant diseases, or education, the anatomical position of the lesion is the most important information. On the other hand, the functional relevance of this anatomical area might be different among different patients, depending, for example, on other lesioned structures as well. Voxel-wise statistics in groups of lesioned patients were able to extend this knowledge [21]. Therefore, lesion mapping is about to find its way in prognostic algorithms in clinical settings and might contribute to prediction of stroke recovery.

To more accurately examine the contribution of white matter damage on motor performance, diffusion-weighted imaging (DWI) strategies have been developed in the last decade, which may exceptionally be integrated into the current clinical routine [18]. DWI-evaluation strategies for motor research are usually limited on tracts connecting areas processing motor control. The standard for hand-motor function is the pyramidal tract running through the posterior limb of the internal capsule (PLIC).

The intactness of the corticospinal tract (CST) can be assessed with axonal or radial diffusivity [22], as well as with fractional anisotropy (FA) [23] within the PLIC. Whereas the usage of axial and radial diffusivity has been criticized [24], the FA is the most robust and most widely applied parameter. FA in the PLIC represents a rather global measure, since tracts from the dorsal premotor cortex (dPMC) and the SMA and the primary motor cortex (M1) and primary somatosensory cortex (S1) and parietal cortex pass through the PLIC.

A decrease in FA in the first days after stroke goes along with the temporal evolution of Wallerian degeneration. Using an ischemic rat model, Wallerian degeneration has been demonstrated to occur during the first days after stroke [25]. In order to assess a robust FA parameter for prognostic considerations, it is recommended to measure DWI not earlier than five days after stroke. Predictability could be improved if DWI is measured after at least two weeks [26].

The intactness of the CST, as tested with diffusion-weighted imaging, has proved very useful for the prediction of hand-motor outcome, especially in more severely affected patients, for example, [27–33]. Parameters assessed with DWI predict the long-term motor outcome better than lesion volume [34]. These parameters can also be used to predict treatment gains in the subacute or chronic stage [35–37].

Besides the corticospinal tract, which has been most frequently assessed, alternate corticofugal fibers and corticocortical connections have recently attracted more attention (for a synopsis, see, e.g., [38]). White matter integrity of noncrossing fibers between M1 and M1 can predict training-induced performance gains in chronic patients [37]. For subacute patients with mild hand-motor impairment, we found a predictive value of M1^il^ to M1^cl^ diffusivity for three- and six-month motor outcome, as tested with the Box and Block test [39]. Especially for tasks requiring bihemispheric resources, such as grasping and transferring objects, these interhemispheric tracts are important, since they enable a bihemispheric coordination of sensorimotor activation [40].

Therefore, structural imaging not only provides stable biomarkers which have become clinically more relevant in predicting motor outcome but also offers enormous potential for developing further parameters to describe the structural intactness of patients in detail.

### 3.2. Activation fMRI: Activation Representation and Network 

#### 3.2.1. Tasks Applied for Activation fMRI after Stroke

Despite the focal damage of a stroke incident, the impact of the lesion leads to local and global changes in brain function. Activation or task-based fMRI has been applied to assess these changes in functional representation after stroke. Two hand-motor tasks are most frequently applied, both allowing a precise control for force and frequency: hand grip using visual feedback of strength [41, 42] and finger sequence using MRI-compatible keyboards [43].

**Figure 3:**
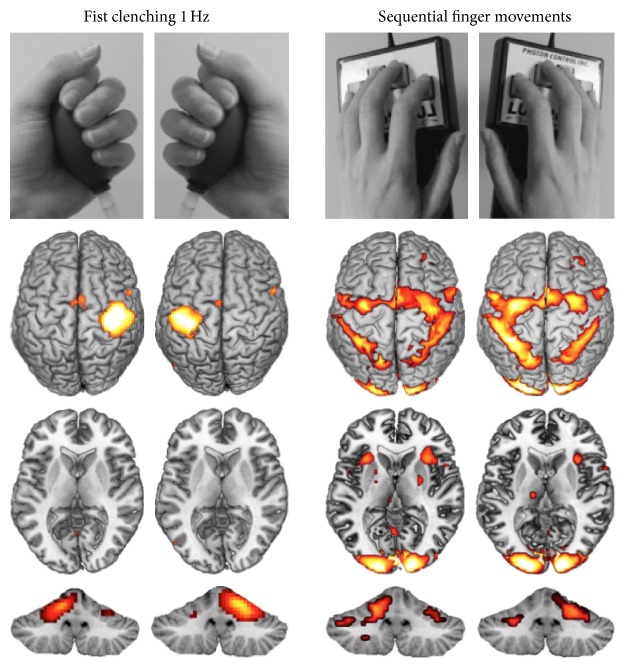
Task characteristics have a crucial impact on fMRI results. Unilateral hand and finger movements performed with left and right hand in a group of 15 right-handed young participants (group results for the premeasurement of the training paradigm reported in [12]). Whereas the fist clenching task shows high lateralization in the cortical and cerebellar representation sites, unilateral finger sequence movements are bilaterally represented. In addition, finger sequences involve basal ganglia and inferior cerebellar hemisphere, at least when performed with the nondominant left hand.

Figure 3 shows typical representation maps for the hand grip modulation and the finger sequence task in a group of healthy young volunteers.

#### 3.2.2. General Findings for Activation fMRI after Stroke

In general, patients showed increased diffuse activation in several areas including motor areas in comparison to healthy controls during different motor tasks. When patients move their affected hand, the lateralization and focus on the contralateral primary sensorimotor cortex during simple unilateral movements (Figure 3) are less expressed than in healthy controls [41]. The increased activation in motor areas of both the damaged hemisphere (ipsilesional) and the unaffected hemisphere (contralesional) has been repeatedly reported (e.g., [44]; meta-analysis by [45]). This increase in activation fMRI in motor areas during simple hand movements often diminishes if recovery is successful. If the increase sustains in later stages, it is often associated with greater motor impairment [41, 46]. The increased use of secondary motor areas can be associated with less functionality of the arm as assessed with accelerometers [47].

In contrast, good motor recovery is related to near-normal activation patterns [48, 49]. One main marker for good recovery seems to be the focused recruitment of the ipsilesional M1 during movement of the affected hand [50–52]. This high recruitment of neural resources of the somatotopic hand representation as a positive sign for well recovered hand-motor function is also characteristic of motor recovery after traumatic brain injury [53].

A prognostic marker for less motor outcome might therefore be assigned to the activation of the contralesional M1 [54] during movements with the affected hand. This hypothesis is supported by a study showing more activity in patients with more severe impairments [55].

Nonetheless, some processes seem to contradict this near to normal hypothesis. The focus on the M1^il^ does not necessarily imply good recovery [56], and even good recovering patients may show M1^cl^ activation [57–60]. The importance of the contralesional hemisphere is not completely understood, but for some patients it may be indicative of involvement of recovered motor function [43]. While lateralization of cortical activation obtained with fMRI is associated with motor impairment in the chronic stage [40], it is not a relevant predictor of change scores resulting from training [61].

In addition, specifically the dorsal premotor cortex (dPMC) has been shown to be profoundly activated in stroke patients [62], and improved motor performance has often been associated with increased dPMC activation in the damaged hemisphere [63]. The dPMC in both hemispheres might have functional significance for patients with partial recovery after stroke [64]. There is also evidence for an enhanced involvement of the ventral premotor cortex (vPMC) [65] and the supplementary motor area (SMA) [63] in restoring motor functions.

#### 3.2.3. Activation fMRI Network Analysis

To elucidate the role of the different regions involved in recovery processes activation, fMRI also enables network analyses to gain information about the interaction of motor areas. There are different methods to assess connectivity between regions, such as dynamic causal modelling (DCM) and structural equation modelling (SEM). An overview of connectivity in stroke networks is provided in [66]. The main results from activation studies are confirmed in network analyses: good recovery is accompanied by network parameters similar to healthy controls. If premotor and supplementary motor areas interact at a lower level with the ipsilesional motor cortex, this decrease is associated with impairment [67–69]. In addition, an inhibitory influence of M1^cl^ to M1^il^ at later stages of recovery is associated with poorer motor outcome [67, 68], which is congruent with the model of suppression of the ipsilesional hemisphere by the contralesional side [70]. Overall, patients that show good recovery have a high integration of M1^il^ in the motor network, for example, [71].

Although network analyses might give more insight into the underlying processes of recovery, they are rarely integrated in prediction analyses. This is mainly due to the hypothesis-driven character of the analyses, focusing only on some aspects of the motor network rather than on its entire complexity. As recovery and the resulting changes in the motor network are highly individual, the same clinical outcome could be driven by different network changes. The methods are therefore promising for assessing individual changes in the motor network over time but are more difficult to apply on inhomogeneous patient groups because different aspects of the motor network are of interest in various recovery courses. In addition, due to the ongoing development of these network analyses, some results are highly dependent on the analysis software [72]. Up to now, more time is needed to establish robust methods before integrating them to prognostic schemes.

#### 3.2.4. Problems with Activation fMRI for Stroke

Whereas the investigation of the functionality of the injured brain during certain motor tasks is promising, there are some difficulties with fMRI protocols.

Since the results obtained in an activation task are dependent on the task and the compliance of the participant to fulfill the protocol, it is crucial to control for task performance; otherwise, performance cannot be distinguished from altered representation. Especially for longitudinal studies, the performance and the effort between the measurements have to be balanced [15]. Performance control during imaging is essential, since movement parameters such as force, amplitude, and frequency are associated with the magnitude of activation fMRI [15, 73, 74]. In addition, especially for large lesions, the question of mirrored movements with the unaffected hand is of high importance, decreasing lateralization of representation to the ipsilesional hemisphere.

Even in healthy subjects, there are major differences in brain activation, depending on the task type (Figure 3), movement patterns involved, task difficulty, or attention. This variance in tasks tested, in addition to the different measures of hand function, makes it more difficult to compare the results of different studies. It is therefore necessary to assess this variance in healthy controls, especially in people of various ages, as the experienced task difficulty and the resulting activation fMRI patterns have been shown to strongly depend on age [75].

Even so, it is often questionable which task depicts the recovery process most accurately. The task has to be accurate enough to capture the impairment effects but simple enough to be carried out by all investigated patients. It is difficult to compare the activation patterns of patients with different severities of stroke. If differences in brain activation only illustrate the task not being executed properly, the usefulness for stroke prediction is low (see, e.g., [76] for compliance of swallowing performance in stroke patients). Therefore, only patients who are able to perform the task can be investigated.

Another problem arises when analyzing movements involving the proximal upper limb (e.g., in aiming tasks), as these increase movement artifacts and are therefore of limited use.

Increased associated head movements in stroke patients who struggle to fulfill the protocol are a general problem, also in other tasks. These head movements exclude a significant number of patients from group analyses, leading to a preselection, and biasing, of patient population.

When examining stroke recovery with fMRI, major problems arise from the interpretation of differences in activation patterns between individuals and the variation within an individual during the recovery course. Therefore, it is difficult to assign the outcome to the activation of a certain region. For example, a diminishing activation of premotor areas in well recovering patients is a good prognostic sign, but patients with more damage to the tracts might profit from dPMC activation which supports motor output. Many factors, such as lesion size, lesion location, age, structural damage, potential for plasticity, previous training experience, and motivation, affect the recovery course and thus the functional activation during motor tasks. In addition, patients in different studies are measured at different time points after stroke. Ward and colleagues have claimed that the depicted changes are more likely a function of recovery than of time [15]. This explains why different patients, even when measured at the same time after stroke, can show different activation patterns.

Activation fMRI analyses can therefore be useful when depicting an individual recovery course over time, as plasticity processes can be observed in direct relation to the motor functions of interest. In examining groups, it is challenging to balance the task requirements over all subjects. Passive tasks may be suited to be used in early stages after stroke, but they do not always reflect the various differences in motor recovery in detail [42].

Therefore, task-based fMRI can be of importance to assess slight differences in compensational areas or lateralization between hemispheres if the patients are preselected according to their ability to fulfill the protocol.

#### 3.2.5. Can Knowledge of Changes in Functional Representation during Short- and Long-Term Training Procedures by Healthy Participants Help to Understand Motor Recovery in Patients after Stroke?

One approach to understanding the processes of motor recovery in patients is the transfer of knowledge of plasticity processes during training in healthy controls. Representational changes after short- and long-term hand-motor training in healthy volunteers show characteristic differences. Short-term training is characterized by an increase in fMRI magnitude in anterior cerebellar hemisphere and the dorsomedial basal ganglia and a decrease in dorsolateral prefrontal cortical representation [77]. In addition to further cortical economization, long-term training is characterized by increased dorsolateral basal ganglia activation and contralateral M1/S1 activation and decreased cerebellar activation [77] (for extremely long trained instrumentalists, see [78]). When performing training protocols developed for stroke patients (arm ability training [79]), healthy young volunteers showed cortical economization in a finger sequence task after two weeks of training for the nondominant upper limb. In a hand grip task with visual feedback, these subjects showed a focused activation pattern in contralateral putamen and ipsilateral anterior cerebellum [12]. In contrast, using the same training strategy to increase hand-motor performance in patients in the subacute stage after stroke, representational changes in visual feedback hand-strength modulation task have only been located in the ventral premotor cortex (vPMC; [42]). Here, the knowledge about fMRI representation of long-term training in healthy volunteers appeared to be of limited value for the training in patients after stroke. In stroke patients there are many reorganization processes, including general recovery processes, task-specific training effects, and compensatory processes [80]. In addition, different lesion locations have different impacts on network disturbances, which might alter short-term and long-term training processes. Overall, processes observed during the recovery of motor ability in patients are difficult to equate with the changes taking place during motor training in healthy volunteers, but assessing training processes in healthy subjects can help to differentiate the various processes involved in stroke recovery.

### 3.3. Resting-State fMRI

In the light of the difficulties of activation of fMRI discussed above, resting-state fMRI (rs-fMRI) seems promising, since it requires little compliance. Rs-fMRI can therefore be conducted in the acute (0–24 hours after stroke onset) to subacute (24 h to 6 weeks after stroke) phase after stroke, comparable to structural MRI [81]. With respect to hand-motor function after stroke, specifically the functional connectivity (FC) of rs-fMRI between cortical motor areas has been described to be associated with motor impairment [82]. Overall, stroke patients with motor impairment show initially decreased interhemispheric M1 connectivity and increased connectivity between ipsilesional M1 and secondary motor areas, particularly in the ipsilesional hemisphere [83].

Indeed, for patients with motor impairment after stroke, an initially decreased rsFC between M1^il^ and M1^cl^ in comparison to healthy age-matched controls is the most consistent finding reported in resting-state studies on stroke patients [69, 82–86]. Previous studies measuring rsFC at the subacute stage found significant associations with motor performance at time of fMRI [82, 84]. This reduced interhemispheric rsFC between the primary motor cortices showed an increase over a period of three months [85] and is associated with motor improvements when increasing up to the level of healthy controls [87]. Park and colleagues [86] investigated rs-fMRI in 12 subacute stroke patients to estimate the value of rsFC for predicting motor outcome. They found a positive association between six-month motor outcome measured with Fugl-Meyer score and rsFC of the M1^il^ with the contralesional thalamus, supplementary motor area (SMA), and medial frontal gyrus.

In contrast, investigating rsFC in the subacute stage, Lindow and colleagues [39] found no predictive value for early or late outcomes. They only observed associations with the motor function at the same time of recovery after mild hand-motor impairment and interpreted the lack of prognostic findings in the highly fluctuating character of rsFC after stroke as previously documented by [85]. The latter authors investigated 31 stroke patients with motor impairment within the first 24 hours and after 7 and after 90 days and found that the reduced interhemispheric sensorimotor (SM1) rsFC normalized over time. Their work is an excellent example of how rsFC can vary after stroke, and this variability may well be the reason why long-term motor outcome prediction is problematic using this measurement.

In the light of different individual recovery curves (see Figure 1), a measurement variable with high predictive value should provide constant parameters in the clinically most interesting subacute phase (which is the case, e.g., with FA measured with DTI). For monitoring the impact of a therapeutic intervention on changes in the motor network, a highly responsive parameter indicating individual changes over time might be more suitable. This has recently been demonstrated with rs-fMRI for monitoring changes induced by transcranial direct current stimulation (TDCS [88]).

### 3.4. Combining Measurements

Each of the methods described here is to some extent suitable for hand-motor outcome prediction, depending on the outcome parameters, time point of measurement, patient group, and so forth. This raises the question of whether different methods might depict associated characteristics or whether they have a different predictive value. Whereas DTI or lesion maps define structural deficits, task-based and task-free fMRIs depict a functionality of the motor network. Therefore, different measures might complement each other.

Some studies included multiple methods to predict motor outcome of stroke patients. DTI and resting-state fMRI have both been assessed to evaluate motor impairment [84, 89] or to predict recovery [39]. In other studies, DTI and activation of fMRI measures also constitute a good combination to detect structural and functional markers for stroke prediction or monitoring of impairment status [61, 90–92]. In addition, transcranial magnetic stimulation (TMS) is frequently used to complement imaging methods [16, 29, 61, 93]. Whereas TMS has a higher positive predictive power, DWI of the pyramidal tract has a higher negative predictive power [94].

The overall results of the various methods were replicated here, but some of the measurements are correlated with each other. For instance, it has been observed that CST damage and rsFC are associated [84, 89, 95]. fMRI measures are also related to the tract damage [40, 92, 96–98]. In general, functional and structural connectivity are often correlated, but functional connectivity can also be present if there is a rather indirect than direct structural connection [99].

Although some potential associations exist, different parameters usually depict different aspects. DTI parameters do not necessarily correlate with TMS measures of tract projections [40] because they may depend on other aspects such as distance of motor neurons stimulated from the scalp (e.g., [100]) or interactions of the motor network.

Similarly, functional parameters such as rsFC and activation of fMRI show no relevant associations [101]. Carter and colleagues found an interesting association between the rsFC and the observed motor outcome [84]. Measuring 23 stroke patients in the subacute stage with resting-state and DTI, they observed that rsFC was only associated with motor outcome when CST damage was low. This study is a good example of the prognostic value of a functional biomarker depending on the structural level.

**Figure 4:**
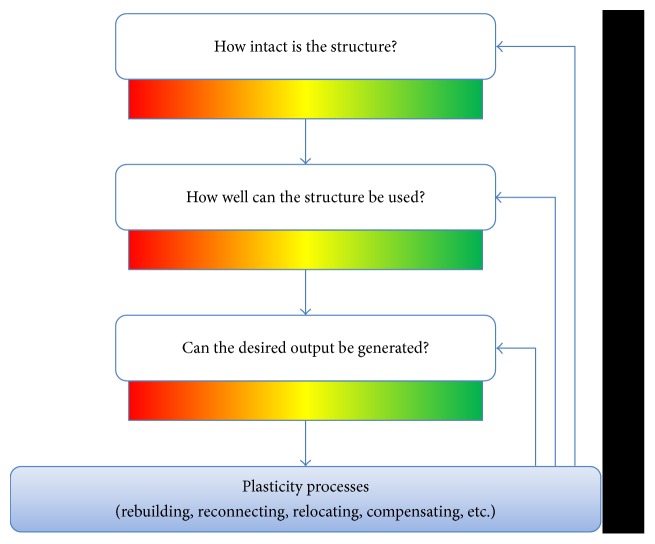
The processes in the brain after stroke can be separated into different hierarchical levels: structural damage (from severe to nearly none) is caused by the stroke incident. Functional recovery potential is usually related to the spared resources of the affected network. The more severe the damage is, the less likely it is to achieve a certain level of functionality. A lack of functionality in turn leads to a poor outcome. Different strategies of the brain, such as compensation with other areas, try to tackle these problems early after stroke. Plasticity processes can start at any of these levels. Note that the intactness and usability of a structure depend on the structural integrity and functionality of various regions and connections, which are important in different ways for generating a certain motor output.

This hierarchical structure needs to be kept in mind when investigating stroke recovery and searching for biomarkers. It is illustrated in Figure 4 that stroke results in structural brain damage; the more brain structures representing motor function are destroyed, the lower the probability to recover motor function is.

After stroke, the primary goal is to regain motor function. Can the desired function be achieved? If the answer is no, the next question is, is there at least a potential for functionality? If the answer is no again, the following question is, is the structure at least as intact as needed for compensatory processes?

These questions are asked by the predicting recovery potential (PREP) algorithm [30] to assess hand-motor outcome after stroke. Biomarkers are used at each level to answer how the patient's status can be described. At the motor output level, the assessment via SAFE score (sum of the shoulder abduction and finger extension) distinguishes patients with already good functionality of the hand and arm from those without. For the latter group, TMS then helps to determine whether there is a potential for functionality and subsequent recovery (i.e., motor-evoked potential (MEP) present/absent). In case of a lack of MEP, a further distinction between patients can be made on a structural level by DWI measurements. The FA parameter in the PLIC described above can give information about the structural damage in terms of an asymmetry index between the affected and unaffected hemisphere. Here imaging methods complement the established assessments to classify patients in groups for therapeutic decisions.

When focusing on motor function, the PREP algorithm makes a meaningful classification. Because in a clinical setting even more fine-grained classifications are needed; the potential for more biomarkers is clear, especially in patients with notable recovery potential, who constitute a rather diverse group.

When evaluating the functionality of a structure, the presence of a MEP is a rather rough measure. It would be interesting to know which regions have retained their functionality. As mentioned before, the restoration of the lesioned motor network is crucial for the recovery of motor performance. If the contralesional hemisphere is involved in simple unilateral movements of the affected hand, prognostic signs are worse. The contralesional hemisphere may interfere with the recovery process of the ipsilesional hemisphere by suppressing the motor output of this hemisphere [70]. Therefore, the functional level could be assessed in more detail, for example, with TMS silent period or resting state determining the balance between hemispheres. If there is a strong interhemispheric imbalance, additional modulations are desirable.

In some groups, a further classification can also be useful, especially regarding therapy decisions. For example, determining the amount of structural damage in other structures apart from the PLIC is valuable in assessing the precise potential for motor recovery or compensation processes. With a more fine-grained classification, better therapy decisions can be made.

It is therefore of utmost importance to investigate certain predefined groups to differentiate them further and to assess multiple parameters early after stroke. Apart from a few exceptions (e.g., [29, 30, 39, 93]), there is a striking lack of prediction studies in acute patients using different modalities. Of course this is due to the high effort when imaging with different measures in a clinical setting. With a stepwise algorithm, however, only a few parameters need to be assessed for each patient, since, for most of the less impaired patients, imaging biomarkers are not necessary. One strategy to cope with the need for decision diagrams is pooling data and establishing large databases. This way, shaping the subgroups may become easier because patients with comparable structural damage can be assessed together. Databases such as PLORAS for the language network are also a promising prototype for motor recovery databases [102]. Specifically, structural imaging or rs-fMRI can complement the existing methods in acute stroke patients, as these methods are easier to implement in the clinical setting than task-based fMRI.

Early measurements can also provide a basis for simulations of how the functionality will probably be affected by certain structural deficits. One approach is implemented in The Virtual Brain (TVB), a novel application for modelling brain dynamics that simulates an individual's brain activity by integrating his own neuroimaging data with local biophysical models [103]. Those biophysical models have to be underpinned with data on the relationship of different measures and an understanding of the processes at different hierarchical levels. However, this understanding still needs to be improved, as most of the methods are still not fully understood, and basic research is necessary to assess all factors influencing the imaging results.

Imaging can add information in the therapeutic decision process if the type of functional deficit can be assessed as early as possible. As mentioned, the hierarchy ends with a certain function on which our assessment is based. It is therefore useful to know which function will be in deficit and needs to be prioritized. Many stroke patients show neuropsychological impairments like aphasia, apraxia, ataxia, neglect, and depression. The relative risk of these impairments might be detected by lesion mapping comparing the location of lesion to probability maps of large data sets of patients functionally investigated. Specifically, those with a high probability of additional impairment need further specific testing. For instance, a patient with neglect needs specific therapy for this concern in addition to motor therapy. In addition, it would be helpful to know and predict the interference among different impairments, for example, with neuropsychological deficits and motor impairments.

Another approach to assessing stroke recovery is apparent from Figure 4: the ongoing process of plasticity applies to every stage of the structure-function hierarchy. As already mentioned, the depicted changes are more likely a function of recovery than of time [15]. Besides the possibility of combining imaging methods, there is the need to measure recovery process longitudinally to assess plasticity processes in detail. This multilayered assessment of individual patients will deepen our understanding of the processes during recovery, which is an important step in the intervention and support with therapy programs.

The mentioned problems regarding the high intersubject variance in methods such as task-based or rest fMRI can be circumvented by using imaging markers as monitoring parameters [15, 88]. In prediction algorithms, imaging can not only help to assess the potential for a certain outcome, but also extend the knowledge predicting the different recovery stages, which may allow us to identify additional factors that contribute to the course of events.

## 4. Conclusion

Procedures with a low level of instruction, low need for patient compliance, short imaging time, and standardized data evaluation strategies are especially promising for both the monitoring and prognosis of hand-motor performance after stroke or brain damage after traumatic brain injury. Over the last years, brain imaging procedures have made a significant step towards becoming a standardized approach, enabling the clinical usage of tools that were previously limited to scientific use. Whereas predictive parameters should be robust and stable over time (such as structural imaging parameters), monitoring needs more sensitive methods for functional changes under intervention (e.g., resting-state fMRI).

In the light of the high prognostic and monitoring effects of nonimaging procedures such as testing motor performance (arm extension) or motor-evoked potentials of hand muscles, the role of the methodologically more challenging imaging procedures is optimally utilized in an algorithm. Currently, the PREP algorithm is the most promising step in this direction. Future extensions will focus on certain subgroups as determined by imaging parameters and assess the best therapy approaches for individual patients. This procedure will help to considerably save resources and optimize neurorehabilitative therapy.
